# Burnout among school teachers: quantitative and qualitative results from a follow-up study in southern Sweden

**DOI:** 10.1186/s12889-019-6972-1

**Published:** 2019-05-29

**Authors:** Inger Arvidsson, Ulf Leo, Anna Larsson, Carita Håkansson, Roger Persson, Jonas Björk

**Affiliations:** 1 Occupational and Environmental Medicine, Lund University, SE-221 85 Lund, Sweden; 2 Centre for Principal Development, Umeå University, Umeå, Sweden; 3 Department of Psychology, Lund University, Lund, Sweden; 4 Centre for Medicine and Technology for Working Life and Society (Metalund), Lund, Sweden

**Keywords:** Exhaustion, Leisure, Psychosocial working conditions, Stress, Work

## Abstract

**Background:**

Teachers are at high risk of stress-related disorders. This longitudinal study aimed to (a) identify which occupational, sociodemographic and life-style factors and self-efficacy at baseline that were of importance for burnout, (b) explore associations between changes in the studied factors versus changes in burnout, and (c) by interviews increase the understanding of perceived job demands among teachers.

**Methods:**

A cohort of 310 Swedish teachers in school-years 4–9 responded to a questionnaire of occupational, sociodemographic and life-style factors, self-efficacy and burnout, at baseline and at follow-up (mean 30 months later). A combined measure with four levels of burnout was crafted, based on exhaustion, cynicism and professional efficacy (Maslach Burnout Inventory-General Survey). Quantitative data were analysed with multiple ordinal regression, and qualitative data were analysed with content analysis of interview responses from a subgroup of the teachers (*n* = 81).

**Results:**

The occurrence of high burnout (level 2 and 3 combined) were similar at baseline and follow-up (14% vs. 15%). However, many teachers fluctuated between the levels of burnout (28% increased and 24% decreased). Burnout at baseline was of importance for change of work or being off duty at follow up. In the multi-exposure model, low self-efficacy [OR 0.42; CI 0.26–0.68] and high job demands [OR 1.97; CI 1.02–3.8] were the strongest explanatory variables. Low self-efficacy remained as the strongest explanatory factor after adjustment for burnout at baseline. Increased job demands during follow-up was associated with an increased level of burnout [OR 3.41; CI 1.73–6.69], whereas increased decision latitude was associated with a decreased level of burnout [OR 0.51; CI 0.30–0.87]. Two major categories of demands emerged in the qualitative analysis; i.e. too high workload and a sense of inadequacy.

**Conclusions:**

A substantial proportion of teachers showed signs of burnout at both occasions. Low self-efficacy and high job demands was of importance for burnout, and changes in burnout was further associated with changes in decision latitude. The results points to the need of actions on individual, organizational and a societal levels.

**Electronic supplementary material:**

The online version of this article (10.1186/s12889-019-6972-1) contains supplementary material, which is available to authorized users.

## Background

According to the Allostasis model [1, 2] and the Cognitive Activation Theory of Stress [3] and similar biomedical mainstream theorizing [4], health is dependent on how well individuals adapt to psychosocial, environmental, and physical challenges. The responses to such challenges to psychological or physiological integrity of the individual is called stress and aims to maintain physiological balance and in extension increase survival and reproductive success. From this perspective it is interesting that the teaching profession stands out and have been identified as one of the most stressful occupations with a potential to cause poor health [5]. In fact, in Sweden teaching is one of the professions with most long-term sick-leave [6] and Johnson et al. [5] compared 26 different occupations and found that teachers scored among the lowest on physical health, psychological well-being and job satisfaction. In addition, high turnover intentions and high sick leave levels have also brought attention to health issues among teachers, especially exhaustion and burnout [7]. Burnout can be described as a psychological syndrome characterized by exhaustion, cynicism/depersonalization and reduced professional efficacy [7]. A previous literature review of studies in different occupational groups has shown that traditional risk factors such as high demands, low job control, high workload, low reward and job insecurity increased the risk for developing exhaustion [8].

The teaching profession entails being subjected to various job demands that often underpin a perception of a heavy workload [5]. However, an increasing time pressure seems to be an international tendency in the teaching occupation [9]. Other examples of job demands are frequent meetings that interfere with preparation time, administrative paper work generated by the management and being subjected to constant reforms and changes that demand re-organization of work and work tasks [10, 11]. The complex work environment and increasing time pressure may also contribute to reduced job control, which is a well-known risk factor for stress (8). Further, teaching is a profession that entails a high degree of face-to-face interaction with pupils, who may show poor behaviour, attitudes, motivation and performance [10, 12]. Other stressors that teachers have to deal with include having to cope with pressure from the parents of the school children and sometimes unrealistic expectations from the society [10]. All these factors may contribute to emotional demands. At the same time, teachers are required to display their own emotions with restraint, i.e. demands of hiding emotions [5].

Given the work conditions outlined above, it is clear that the teacher’s personal resources are important to counter the effects of a stressful work situation. One personal resource of importance for performance is self-efficacy. Self-efficacy is often considered important as it concerns the appraisal of one’s capabilities to successfully carry out a particular course of action [13]. According to Taris and Schaufeli [14] self-efficacy could act as a personal resource by influencing the perceptions of work demands and resources; which in extension may affect commitment, well-being and health. A previous study showed that self-efficacy buffered the demands-strain relationship among teachers [15]. In another study it was shown that self-efficacy was significantly negatively associated to the depersonalisation and emotional exhaustion dimensions of burnout, and significantly positively associated to the personal accomplishment dimension [16].

Another important resource and aspect of work is access to social support [17, 18]. For example, Littrell, Billingsley and Cross [19] showed that when principals are emotionally supportive and provide informational support, teachers report greater job satisfaction, occupational commitment and health. Yet another occupational factor that may have importance for the teachers’ well-being is the working conditions during computer work that can bring both physical and mental demands [20].

Besides work stress, life-style factors such as insufficient time for personal relaxation [21] and lack of energy for domestic work [22], have been shown to contribute to the development of stress and burnout, while physical activity may be a protective factor [23].

In the present study, we build on an earlier study of 490 Swedish school-teachers [24], in which we explored cross-sectional associations between occupational and sociodemographic factors, life-style, self-efficacy and burnout. In that study we used a combined burnout measure based on the three dimensions exhaustion, cynicism and professional efficacy, which constituted the outcome of burnout in four levels of increasing seriousness (level 0–3). We observed that the perception of low self-efficacy, high job demands, poor leadership and teaching in higher grades were the factors that were most strongly associated with high burnout at baseline. As high job demands and low self-efficacy were the factors most strongly associated with burnout at baseline it is of interest to investigate to what extent these or other factors are of importance for burnout levels across time. By including sociodemographic and lifestyle factors, we hope to control for confounding when assessing the impact of various aspects of the job environment. Therefore, in the present study we present the results from longitudinal analyses using data from a follow-up survey conducted 2–3 years later.

### Aim

Our study had three distinct aims:

1. To identify the relative importance of occupational, sociodemographic and life-styles factors and self-efficacy among teachers at baseline, for burnout two to three years later, with a special interest in job demands and self-efficacy.
2. To investigate how changes in occupational, sociodemographic and life-style factors and self-efficacy during the follow-up period are associated with burnout at follow-up.
3. To increase the understanding of the perception of job demands among teachers.

## Methods

### Study design and participants

The present study participants were part of a parallel study of work related musculoskeletal disorders that entailed teachers, nurses and sonographers [25]. The present two-wave longitudinal study utilised a non-random sampling strategy that targeted these three specific occupational groups characterized by either physical (nurses and sonographers) or by mental workload (teachers).

The participants in the present study sample of teachers responded to a questionnaire that included questions on occupational, sociodemographic and life-style factors, self-efficacy and burnout [24, 25]. The questionnaire was administered at baseline (2010–2012) and at follow-up (2012–2014) with a follow-up period of mean 30 months (SD 3.0 months). The length of follow-up periods did not differ noticeably between the genders or between teachers in different year of compulsory school.

The baseline questionnaire was directed to 769 teachers employed at 50 compulsory schools across seven Swedish municipalities, whereof 490 teachers (134 men and 356 women) participated in the study. In each school, all teachers educating children in theoretical subjects in school years 4–9 (aged 10–15 years) were invited. A further inclusion criterion was work at least 50% of fulltime during a period of at least 3 months before fulfilling the baseline questionnaire.

A subgroup of the teachers, i.e. all teachers at three of the participating schools, were invited to participate in an on-site clinical examination and an interview about their working conditions. Out of 89 invited teachers 81 accepted participation, while eight teachers declined. These interview-responses from the teachers were included in the qualitative part of the present study.

Out of the 490 teachers at baseline, 310 participated at follow-up. The response rate was 63% (65% for the women and 60% for the men). High burnout (level 2 and 3 combined) at baseline was more frequent among non-participants (17%) than among participants (14%; Table 1). The drop-outs from baseline to follow-up was a heterogeneous group: the frequency of high burnout at baseline was 37% among the teachers who were off duty/changed work at follow-up vs. 14% among the participants (*p* = 0.055; Fisher’s exact test). In contrast, the frequency of high burnout at baseline was low among teachers who retired during the follow-up period (4%).

**Table 1:** Distribution of participants across increasing levels of burnout (0 to 3) in the total study sample at baseline (*N* = 490) as well as for the participants that dropped out of the study between baseline and follow-up (i.e., “drop-outs”)

		Levels of burnout at baseline			
	All	Level 0	Level 1	Level 2	Level 3
	N	N (%)	N (%)	N (%)	N (%)
Included in the follow-up study	310	165 (53)	102 (33)	30 (10)	13 (4)
Women	230	120 (52)	78 (34)	22 (10)	10 (4)
Men	80	45 (56)	24 (30)	8 (10)	3 (4)
Drop-outs, all	180	83 (46)	67 (37)	23 (13)	7 (4)
Non responders*	91	39 (43)	35 (38)	15 (16)	2 (2)
Off duty/change of work	24	10 (42)	5 (21)	6 (25)	3 (12)
Retired	43	25 (58)	16 (37)	2 (4)	0
Parental leave	11	4 (36)	5 (46)	0	2 (18)
Missing outcome-data at follow-up	11	5 (46)	6 (54)	0	0

*Three persons could not be reached, two had emigrated

### Measures

#### Burnout

The 16-item version of the Maslach Burnout Inventory-General Survey (MBI-GS [26, 27] was used. The items in MBI-GS cover three dimensions: exhaustion (5 items), cynicism (5 items), and professional efficacy (6 items). All items were responded to on a 7-point scale: 0 = “never”; 1 = “a few times a year or less”; 2 = “once a month or less”; 3 = “a few times a month”; 4 = “once a week”, 5 = “a few times a week” and 6 = “every day”. The mean score for each dimension was calculated and used as an outcome in unadjusted group comparisons. In addition, we applied a previously used supplementary scoring procedure [24] that entailed making a dichotomous classification of each item according to the linguistic meaning of the response alternatives. Each item was therefore dichotomized into 0 = “low” or 1 = “high” in relation to a cut-off score of 4 = “once a week”. Accordingly, to be classified as a burnout case at least three of the five dichotomized items had to be high on the exhaustion and cynicism dimensions. For the six professional efficacy items, at least three had to be high to be classified as having burnout in terms of low professional efficacy. In addition, an individual-level composite measure of the three burnout dimensions was created by combining the dichotomized responses into four ordered categories: 0 = subjects reporting low exhaustion, low cynicism and high professional efficacy (referents); 1 = subjects reporting either high exhaustion or high cynicism or low professional efficacy (one out of the three dimensions); 2 = subjects reporting high exhaustion and/or high cynicism and/or low professional efficacy (two out of the three dimensions); and, 3 = subjects reporting high exhaustion and high cynicism and low professional efficacy (all three dimensions).

#### Ergonomic working conditions

One study-specific item assessed to what extent the participants were satisfied with their computer work stations. The item read: *“*Are you satisfied with the *computer work-station arrangements?”* and was responded to on a five-point scale: 1= “yes, very satisfied (can work comfortably)”, 2 = “yes, rather satisfied”, 3= “neither satisfied nor dissatisfied”, 4 = “no, rather dissatisfied”, 5 = “no, very dissatisfied (uncomfortable/strenuous work)”.

#### Psychosocial working conditions

The psychosocial conditions at work were in part assessed with a Swedish version of the Job Content Questionnaire (JCQ) [28, 29] that covered three dimensions: Job demands (9 items), Job control (9 items) and Job support (8 items). The items were responded to on a four-point scale, indicating the level of agreement with various statements about conditions at work (1 = totally disagree, 2 = disagree, 3 = agree, 4 = totally agree). The mean value in each dimension was calculated for each individual, and the mean scores were used as continuous measures in the analysis [28, 29]. Higher scores indicated higher demands, more control, and better support.

The JCQ was supplemented with 18 items from the Copenhagen Psychosocial Questionnaire [30] that assessed: emotional demands (3 items), demands on hiding emotions (2 items), sensory demands (5 items), and leadership (8 items). All questions were answered on a five-point scale. The mean score in each dimension was calculated for each individual and used as a continuous variable [30]. Higher scores indicate higher demands and better leadership.

#### Self-efficacy

General self-efficacy was assessed with three items [31]. The items were formulated as statements and read: “You can deal with most unexpected events”, “You can solve most problems if you really want to” and “Irrespective of what is going on in your life, you feel that you can handle it”. All items had five response categories: 1 = “never/ hardly ever”, 2 = “seldom”, 3 = “sometimes”, 4 = “often”, and 5 = “always”. The mean score (range 1–5) was calculated for each individual and used as a continuous variable in the analysis. Higher scores indicated greater self-efficacy.

#### Sociodemographic and life-style factors

Information was collected about gender, age and marital status. Further, *o*ne study-specific item assessed *personal recovery* and read: “How much of your leisure time do you normally use for personal recovery?” The item was responded to on a 5-point scale: 1 = hardly any time at all; 2 = < 1 h/day, 3 = 1 h/day; 4 = 2 h/day; 5 = 3 h/day and 6 = ≥4 h/day. Another study-specific item assessed *domestic work* and read: “How many hours a week, do you normally work at home doing cleaning, gardening, cooking, etc.?” The item was responded to on a 5-point scale: 1 = 0–2 h/week; 2 = 3–10 h/week; 3 = 11–20 h/week; 4 = 21–30 h/week and 5 = ≥ 31 h/week). Exercise was assessed by asking about the frequency of *physical exercise* (0 = never; 1 = occasionally; 2 = once a week; 3 = 2–4 times/week; 4 ≥ 5 times/week).

### Interviews of the teachers

The interviews of the subgroup of 81 teachers included four open questions: “Which favourable and unfavourable work conditions do you perceive?”, “Which work tasks do you perceive as ergonomically stressful?” and “Do you have any suggestions of improvements of the work environment at your workplace?” The interview guide is given in Additional file 1. Each of the interviews lasted for about 10 minutes. The teachers answered the open questions and the interviewer registered their responses by taking field notes (and thus performed the first condensation of the answers). In the present study, the answers of the question “unfavourable working conditions” were selected and further analysed with a focus on answers associated with job demands.

### Quantitative analyses

All statistical analyses of quantitative data were performed with the IBM SPSS software, version 24 (IBM Corp.). *P*-values ≤0.05 (two-tailed) were considered as statistically significant.

Analyses of differences between follow-up and baseline were made for the dimensions exhaustion, cynicism and professional efficacy, with the non-parametric Wilcoxon Signed Ranks Test.

The participants at baseline (*n* = 490) were stratified into low/high demands (dichotomized by the median value 2.9) and low/median/high self-efficacy (scores < 3, 3 and > 3).

Among the teachers who participated at both baseline and follow-up (*n* = 310), the Jonkheere-Terpstra test for trend was used to examine occupational, sociodemographic and life-style factors at baseline, across the four ordered levels of increased burnout at follow-up (Additional file 2: Table S1).

Odds ratios (ORs) and 95% confidence intervals (CIs) for the importance of burnout (level 0–3) at follow-up were first estimated in single-exposure ordinal regression models for all variables (occupational, sociodemographic and lifestyle factors and self-efficacy) at baseline. We are using the cumulative odds model with location parameters only, which estimates average odds ratios (ORs) of all possible dichotomizations of the ordinal response variable.

In the next step, ORs for levels of burnout at follow-up were estimated using multi-exposure ordinal regression, for variables with single-exposure *p*-values < 0.3. In the multi-exposure ordinal regression models, the psychosocial dimensions job demands, job control and job support from job content questionnaire were chosen as explanatory factors prior to emotional demands, demands of hiding emotions and leadership from the Copenhagen Psychosocial Questionnaire. This decision was based on the risk for conceptual overlap and a substantial Spearman correlation coefficient (R^S^) with the dimensions in Job Content Questionnaire (job demands - emotional demands R^S^ 0.48; Job demands – leadership R^S^ 0.47; leadership – job support R^S^ 0.69). In the last step, by adjusting for the level of burnout at baseline we tried to quantify how much of the different explanatory factors for burnout at follow-up that were due to associations with burnout that were present already at baseline.

Using single- and multi-exposure ordinal regression with burnout at follow up (level 0–3) as dependent variable, we also analysed how changes between baseline and follow-up in occupational and life-style factors and self-efficacy (calculated for each individual by subtracting the baseline scores from the follow-up scores) were associated with (a) the level of burnout at follow-up, and (b) changes in the level of burnout between baseline and follow-up. The latter analysis was performed by adjusting the multi-exposure model for the level of burnout at baseline.

### Qualitative analysis

Qualitative data from the interviews were analysed by content analysis [32], and with simple frequency counts of the categories that emerged from the data. First, all the notes were transcribed into a word document, and read to get a sense of the whole. In the next step meaning units were extracted, condensed and labelled with a code. Next all codes were interpreted and compared for differences and similarities and two categories emerged.

## Results

Basic characteristics for the participants at baseline are presented, stratified for the two variables that stood out in the previous cross-sectional analysis [24], that is, self-efficacy and job demands, in Table 2**.** Generally, while high job demands co-occurred with a number of other work-related dimensions, self-efficacy seemed to be more of a personal characteristic and less associated with other psychosocial dimensions. The continuous variables job demand and self-efficacy were weakly correlated (rho = 0.09, **not in table**).

**Table 2:** Occupational-, sociodemographic-, life-style factors and self-efficacy at baseline, among 490 teachers (356 females and 134 males), stratified into low/high demands, and low/median/high self-efficacy

		Job demands		Self-efficacy		
*Characteristics at baseline*	*Scale*	Low (*N* = 272)	High (*N* = 214)	Low (*N* = 101)	Median (*N* = 214)	High (*N* = 172)
*Year of compulsory school*						
year 4–6; n (%)		93 (34)	57 (27)	33 (33)	76 (35.5)	44 (26)
year 7–9; n (%)		179 (66)	157 (73)	68 (67)	138 (64.5)	128 (74)
Seniority; mean (SD)	*years*	17 (12)	17 (12)	18 (12)	17 (12)	17 (12)
Complaints on computer						
workstation arrangements; mean (SD)	*1-5* ^*a*^	2.8 (1.0)	3.4 (1.1)	3.2 (1.2)	3.1 (1.0)	3.0 (1.1)
Job demands; mean (SD)	*1-4* ^*a*^	n.a.	n.a.	3.0 (0.4)	2.9 (0.4)	2.9 (0.4)
Job control – decision latitude; mean (SD)	*1-4* ^*b*^	3.1 (0.4)	3.0 (0.5)	3.0 (0.4)	3.1 (0.4)	3.1 (0.5)
Job control - skill discretion; mean (SD)	*1-4* ^*b*^	3.4 (0.3)	3.4 (0.3)	3.3 (0.3)	3.4 (0.3)	3.4 (0.3)
Job support from manager; mean (SD)	*0-4* ^*b*^	2.8 (0.5)	2.4 (0.6)	2.5 (0.6)	2.6 (0.5)	2.6 (0.7)
Job support from collegues; mean (SD)	*0-4* ^*b*^	3.1 (0.4)	3.0 (0.5)	3.0 (0.5)	3.1 (0.4)	3.1 (0.4)
Emotional demands; mean (SD)	*0-4* ^*a*^	2.5 (0.7)	3.1 (0.6)	2.9 (0.7)	2.8 (0.7)	2.7 (0.8)
Demands of hiding emotions; mean (SD)	*0-4* ^*a*^	1.5 (0.8)	1.9 (0.7)	1.8 (0.8)	1.7 (0.8)	1.6 (0.8)
Leadership; mean (SD)	*0-4* ^*b*^	2.3 (0.7)	1.7 (0.80)	1.9 (0.9)	2.1 (0.8)	2.1 (0.9)
Self-efficacy; mean (SD)	*1-5* ^*b*^	4.1 (0.5)	4.0 (0.5)	n.a.	n.a.	n.a.
*Gender*						
Men; n (%)		85 (31)	47 (22)	24 (24)	57 (27)	51 (30)
Women; n (%)		187 (69)	167 (78)	76 (76)	157 (73)	121 (70)
*Marital status*						
Married/cohabit; n (%)		224 (85)	179 (84)	79 (80)	187 (89)	139 (83)
Single; n (%)		40 (15)	33 (16)	20 (20)	24 (11)	28 (17)
Age; mean (SD)	*years*	48 (11)	47 (11)	48 (12)	48 (11)	47 (11)
Personal relaxation time; mean (SD)	*1-6* ^*b*^	3.7 (1.3)	3.3 (1.3)	3.7 (1.4)	3.5 (1.3)	3.5 (1.3)
Domestic work; mean (SD)	*1-5* ^*a*^	2.8 (0.9)	2.9 (0.9)	2.75 (1.1)	2.9 (0.8)	2.85 (0.9)
Physical exercise; mean (SD)	*0-4* ^*b*^	2.8 (1.1)	2.55 (1.1)	2.8 (0.8)	2.6 (1.1)	2.7 (1.1)

^a^ Higher scores indicate a more unfavourable situation

^b^ Higher scores indicate a more favourable situation

### Changes in burnout from baseline to follow up

The mean exhaustion score increased between baseline and follow-up (2.8 vs. 3.0, respectively; *p* = 0.05), while the mean cynicism and professional efficacy scores did not differ (1.6 vs. 1.7; *p* = 0.26 and 5.0 vs. 5.1; *p* = 0.12, respectively). The patterns of the original dimensions of MBI-GS (i.e. exhaustion, cynicism and professional efficacy) across the four levels of burnout are shown in Table 3.

**Table 3:** MBI exhaustion, cynicism and professional efficacy in the total study sample at follow-up, stratified by the four levels of burnout

			Burnout at follow-up				
MBI dimensions at follow-up; mean (SD)	Scale	N	All	Level 0	Level 1	Level 2	Level 3
			*N* = 310	*N* = 143	*N* = 119	*N* = 38	*N* = 10
*Exhaustion*	0–6	310	3.0 (1.5)	1.9 (0.9)	3.7 (1.2)	4.5 (0.8)	5.0 (0.5)
*Cynicism*	0–6	310	1.7 (1.3)	1.1 (0.9)	1.7 (0.9)	3.5 (1.3)	4.5 (0.7)
*Professional efficacy*	0–6	310	5.1 (0.7)	5.4 (0.5)	4.9 (0.7)	4.6 (0.8)	3.8 (0.5)

**Fig. 1:**
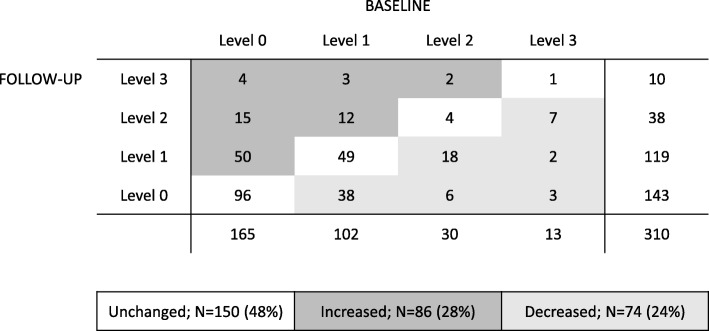
Levels of burnout at baseline and at follow-up. Cross tabulation of the number of participants at baseline and follow-up, distributed across the four levels of burnout

The frequency of high burnout (level 2 and 3) were similar at baseline and follow-up (14% vs. 15%). However, about half of the teachers (48%) fluctuated between the different levels of burnout (Fig. 1**).** Increasing levels of burnout was found in 28% of the teachers, while 24% of the teachers reported decreased burnout and some of them were recovered at follow-up (level 0). About one third (31%) reported low burnout (level 0) at both occasions.

### Single-exposure models of risk factors for burnout at follow-up

Description of all occupational, sociodemographic and life-style factors and self-efficacy at baseline, stratified by the four levels of burnout at follow-up, are given in Additional file 2: Table S1.

In single-exposure models, several factors at baseline was of importance for the level of burnout at follow-up (Table 4). Burnout at baseline and the perceptions of high job demands, high emotional demands, high demands of hiding emotions and much complaints on the computer workstation arrangements were all significant explanatory factors for high burnout at follow-up. High support from manager, high self-efficacy and perceived good leadership at baseline explained lower levels of burnout. The same was true for much physical exercise and much time for personal relaxation. Year of compulsory school, decision latitude, skill discretion, support from colleagues, gender, age, seniority, marital status and the amount of household work were not of significant importance for burnout at follow-up.

**Table 4:** Single- and multi-exposure ordinal regression models in the total study sample of associations between occupational, sociodemographic- and life-style factors and self-efficacy *at baseline* and levels of burnout *at follow-up* (level 0 = reference)*,* estimated with Ordinal Regression, with p-values, odds ratios (OR) and 95% confidence intervals (CI). In the last step, the multi-exposure model was adjusted for burnout (level 0–3) at baseline

			Burnout at follow-up (level 0–3)					
	*Scale*	N	Single-exposure models		Multi-exposure model		Multi-exposure model, adjusted	
*Characteristics at baseline*			p	OR (CI 95%)	p	OR (CI 95%)	p	OR (CI 95%)
Year of compulsory school (year 7–9)		310	0.08	1.51 (0.96–2.38)	0.52	1.18 (0.71–1.97)	0.68	1.11 (0.66–1.86)
Seniority; mean (SD)	*years*	310	0.10	0.98 (0.97–1.00)	0.24	0.99 (0.97–1.01)	0.18	0.99 (0.97–1.01)
Complaints on computer								
workstation arrangements; mean (SD)	*1–5*	306	0.01	1.28 (1.05–1.57)	0.45	1.09 (0.87–1.36)	0.42	1.10 (0.88–1.37)
Job demands; mean (SD)	*1–4*	308	< 0.001	3.08 (1.80–5.26)	0.04	1.97 (1.02–3.8)	0.205	1.56 (0.78–3.11)
Job control – decision latitude; mean (SD)	*1–4*	310	0.57	0.87 (0.55–1.39)		-^a^		-^a^
Job control - Skill discretion; mean (SD)	*1–4*	310	0.10	0.54 (0.27–1.11)	0.69	0.85 (0.38–1.90)	0.86	0.93 (0.41–2.09)
Job support from manager; mean (SD)	*1–4*	310	< 0.001	0.44 (0.29–0.67)	0.11	0.65 (0.39–1.10)	0.15	0.68 (0.40–1.14)
Job support from collegues; mean (SD)	*1–4*	308	0.12	0.67 (0.41–1.11)	0.39	0.78 (0.45–1.38)	0.45	0.80 (0.45–1.41)
Emotional demands; mean (SD)	*0–4*	310	< 0.001	1.79 (1.30–2.46)		-^b^		-^b^
Demands of hiding emotions; mean (SD)	*0–4*	310	0.007	1.47 (1.11–1.95)		-^b^		-^b^
Leadership; mean (SD)	*0–4*	310	< 0.001	0.59 (0.45–0.77)		-^b^		-^b^
Self- efficacy; mean (SD)	*1–5*	309	< 0.001	0.38 (0.24–0.60)	< 0.001	0.42 (0.26–0.68)	0.02	0.53 (0.31–0.90)
Gender (women)		310	0.69	0.91 (0.56–1.46)		-^a^		-^a^
Marital status (single)		306	0.86	0.95 (0.50–1.80)		-^a^		-^a^
Age, mean (SD)	*years*	310	0.33	0.99 (0.97–1.01)		-^a^		-^a^
Personal relaxation time; mean (SD)	*1–6*	304	0.001	0.72 (0.60–0.87)	0.12	0.86 (0.71–1.04)	0.16	0.87 (0.72–1.06)
Household work; mean (SD)	*1–5*	306	0.42	1.11 (0.86–1.43)		-^a^		-^a^
Physical exercise; mean (SD)	*0–4*	307	0.001	0.72 (0.60–0.87)	0.12	0.85 (0.69–1.04)	0.12	0.85 (0.69–1.04)
Burnout at baseline	*overall*	310	< 0.001				0.17	
	*Level 0*	165		1				1
	*Level 1*	102		2.06 (1.28–3.31)				1.56 (0.91–2.65)
	*Level 2*	30		3.67 (1.80–7.57)				2.02 (0.90–4.55)
	*Level 3*	13		9.68 (3.25–28.8)				3.05 (0.80–11.5)

^a^ Not included in the multi-exposure model, due to a p-value ≥0.3 in the single-exposure model

^b^ Not included in the multi-exposure model, due to a high collinearity with the dimensions job demands and/or job support

### Multi-exposure models for burnout at follow-up

The results indicated that low self-efficacy [OR 0.42; CI 0.26–0.68] and high job demands [OR 1.97; CI 1.02–3.8] at baseline were the strongest explanatory factors of high burnout at follow-up (Table 4). An OR = 1.97 associated with job demands means that the odds of scoring high rather than low on the burnout scale during follow up is 1.97 higher on average among teachers with one unit higher job demand at baseline (e.g. 3 rather than 2). Note that the OR is an average estimate across all possible dichotomizations (high vs. low) of the ordinal burnout scale.

When the model was adjusted for the level of burnout at baseline, low self-efficacy remained as the variable with most importance (Table 4). The impact of burnout at baseline decreased, and the confounding was mostly due to self-efficacy and only to a minor extent due to job demands (not in tables).

### Changes in occupational, sociodemographic and life-styles factor and self-efficacy in relation to burn-out at follow up

For the total study sample, there were only minor numerical differences in mean values between the occupational, sociodemographic and life-styles factor and self-efficacy at baseline and follow-up (Table 5). However, as shown by the standard deviations, there were large individual variations for all the investigated factors. In the single-exposure models increased job demand, decreased decision latitude and increased emotional demands were associated with a high level of burnout at follow-up.

**Table 5:** Changes between baseline and follow-up in occupational, sociodemographic^a^- and life-style factors and self-efficacy in the total study sample (for each individual calculated as follow-up – baseline); mean values and standard deviation (SD). Single- and multi-exposure models between the changes in the potential risk factors and burnout *at follow-up* (levels 0–3), estimated with Ordinal Regression, with *p*-values, odds ratios (OR) and 95% confidence intervals (CI). In the last step, the multi-factor model was adjusted for burnout (level 0–3) at baseline, thereby estimating change versus change associations

				Burnout at follow-up (levels 0–3)					
	*Scale*	N	Follow-up - baseline	Single-exposure models		Multi-exposure model		Multi-exposure model, adjusted	
*Factors at baseline*			Mean (SD)	p	OR (CI 95%)	p	OR (CI 95%)	p	OR (CI 95%)
Complaints on computer									
workstation arrangements; mean (SD)	*1–5*	306	0.0 (1.2)^b^	0.40	1.08 (0.90–1.31		-^d^		-^d^
Job demands; mean (SD)	*1–4*	308	0.0 (0.4)^b^	< 0.01	2.15 (1.21–3.81)	0.02	2.09 (1.12–3.92)	< 0.001	3.41 (1.73–6.69)
Job control – decision latitude; mean (SD)	*1–4*	310	0.0 (0.5)^c^	< 0.01	0.53 (0.34–0.83)	0.01	0.48 (0.29–0.81)	0.01	0.51 (0.30–0.87)
Job control - Skill discretion; mean (SD)	*1–4*	310	0.0 (0.3)^c^	0.06	0.49 (0.23–1.03)	0.17	0.56 (0.24–1.28)	0.10	0.49 (0.21–1.15)
Job support from manager; mean (SD)	*1–4*	310	0.0 (0.5)^c^	0.20	0.77 (0.52–1.15)	0.54	1.15 (0.73–1.81)	0.85	1.04 (0.66–1.66)
Job support from collegues; mean (SD)	*1–4*	308	0.0 (0.4)^c^	0.26	0.74 (0.44–1.25)	0.34	0.75 (0.42–1.35)	0.22	0.69 (0.38–1.26)
Emotional demands; mean (SD) ^d^	*0–4*	310	−0.1 (0.6)^b^	0.04	1.42 (1.01–2.0)		- ^e^		- ^e^
Demands of hiding emotions; mean (SD) ^d^	*0–4*	310	0.0 (0.8)^b^	0.07	1.29 (0.98–1.71)		- ^e^		- ^e^
Leadership; mean (SD) ^d^	*0–4*	310	0.0 (0.8)^c^	0.48	0.91 (0.69–1.19)		- ^e^		- ^e^
Self- efficacy; mean (SD)	*1–5*	309	0.0 (0.5)^c^	0.06	0.62 (0.38–1.01)	0.26	0.73 (0.42–1.26)	0.05	0.57 (0.32–1.01)
Personal relaxation time; mean (SD)	*1–6*	304	0.0 (1.2)^c^	0.86	0.98 (0.83–1.17)		- ^d^		-^d^
Household work; mean (SD)	*1–5*	306	−0.1 (0.9)^b^	0.06	1.27 (0.99–1.63)	0.23	1.18 (0.90–1.53)	0.11	1.25 (0.95–1.65)
Physical exercise; mean (SD)	*0–4*	307	0.0 (1.1)^c^	0.40	1.08 (0.90–1.31)		- ^d^		-^d^
Burnout at baseline	*overall*	310						< 0.001	
	*Level 0*	165							1
	*Level 1*	102							2.65 (1.55–4.53)
	*Level 2*	30							8.79 (3.73–20.7)
	*Level 3*	13							23.0 (6.53–80.7)

^a^Age, seniority, changes in year of compulsory school (only 7 participants) and changes in marital status (11 participants) were not included in the analysis

^b^Positive values means worse conditions at follow up

^c^Positive values means better conditions at follow-up

^d^Not included in the multi-exposure model, due to a p-value ≥0.3 in the single-factor models

^e^Not included in the multi-exposure model, due to a high collinearity with the dimensions job demands and/or job support

When adjusting for the level of burnout at baseline, i.e. in analysis how *changes* in occupational, sociodemographic and life-styles factors and self-efficacy between baseline and follow-up were associated with *changes* in burnout (Table 5), a statistically significant co-variation was found for job demands and decision latitude: Increased job demands was associated with an increased level of burnout [OR 3.41; CI 1.73–6.69], whereas increased decision latitude was associated with a decreased level of burnout [OR 0.51; CI 0.30–0.87]. Further, the level of burnout at baseline was of importance.

### Interview of the teachers

In the content analysis of the individual interviews of a subgroup of the teachers (*n* = 81) two major categories of demands emerged: Too high workload and a sense of inadequacy.

#### Too high workload

Approximately half of the group of the interviewed teachers (*n* = 42), stated that an increase of administrative work tasks contributed to the high workload. Thus, the high workload was not attributed to the teaching itself but rather to the continuous increase of new demands that was added without removing other work tasks. This may be exemplified by the following field-note that quotes one teacher:

"It is much too much that is laid on us. It is outrageous that they put on work tasks without telling what should be removed. Many say they will not be able to continue working." (Teacher in school-year 4-6).

Many teachers stated that the conditions and the increasing amount of administration had a negative impact on the planning of lessons. For example, the teachers reported that they had to deal with new technological systems, new requirements for long term educational planning to align the teaching with the goals, new requirements for grading and assessment of students’ results and extensive individual development plans for each student. Other teachers emphasized a general lack of resources, resulting in too large student groups. Another source of demands put forward by the teachers concerned how teacher absenteeism was dealt with. With respect to this, some teachers explained that there were no substitutes and thus the teachers must fill in for each other. This was perceived to cause unplanned changes in the schedule, a higher work load and limited possibilities to take a break during the working day.

#### Sense of inadequacy

Circa 40% of the teachers (*n* = 31) in the interviews expressed a sense of inadequacy. According to the teachers, the sense of inadequacy was due to too many work tasks and time pressure combined with a feeling of not being able to do a good job and achieve their pedagogical goals. Many teachers perceived that they did not have time enough to prepare the teaching activities, to interact with the pupils or to meet the needs of pupils with special needs. Altogether, this sense of inadequacy was perceived to lead to frustration, stress and being forced to prioritize different students. This may be exemplified by the following field-note that quotes one teacher:

“Being insufficient is frustrating. You have to choose which students you can devote yourself to” (Teacher in school-year 7–9)

## Discussion

### Principal findings

In the single-exposure models, several of the studied factors at baseline, including the level of burnout, were of importance for the level of burnout at follow-up. In the multi-exposure model, among the occupational and personal factors, low self-efficacy and high job demands were the strongest explanatory variables. These two factors were weakly correlated; and thus appear to be independently of importance for burnout.

Changes in job demands and decision latitude scores between baseline and follow-up were associated with changes in the levels of burnout between baseline and follow-up. Increases in job demand scores was associated with an increase in burnout whereas an increase in decision latitude scores was associated with a decrease in burnout.

The content analysis of the individual interview responses, which were provided from a subgroup of the participants, identified that work demands could be classified into two major, but partly intertwined, categories: Too high workload and a sense of inadequacy. A majority of the teachers perceived that an increasing amount of administrative tasks contributed to an increased workload and had a negative impact on the available time for planning of lessons. Many teachers experienced that time pressure combined with a feeling of not being able to do a good job lead to a sense of inadequacy.

Regarding the underlying dimensions of the burnout measure, the mean values in the dimension exhaustion increased between baseline and follow-up, while the mean values in cynicism and professional efficacy did not differ significantly. The frequency of high burnout (level 2 + 3) was similar at baseline and at follow-up (14% vs. 15%, respectively). However, we observed a large fluctuation on the individual level. About one fourth of the teachers reported an increased level of burnout at follow-up, while another fourth reported a reduced level.

Additionally, among the drop-outs from our study, burnout at baseline was associated with being off duty/changing work at follow up. This may be an indicator of that teachers with high burnout were more likely than those with lower levels of burnout, to change job or find other alternatives in order to avoid unfavourable working conditions.

### Strengths and weaknesses of the study

There are several strengths but also several limitations in this study that warrant attention before we reach to the conclusions. To begin with, a strength is the longitudinal study design and that we used a common measure of burnout (i.e., MBI-GS) as well as common and tested indicators for occupational, sociodemographic and life-style factors and self-efficacy. Another advantage is the use of the burnout-measure both in the standard way (i.e. calculating a mean score in the three dimensions exhaustion, cynicism and professional efficacy), and as a combined measure of the three dimension on an individual level. Since the original three dimensions were incrementally more unfavorable reported through the increasing levels of burnout, we considered the combined measure as a relevant outcome in the analysis, and a major strength of the study. Further, that we approached teachers in 50 schools across seven municipalities in the south of Sweden is also a strength of the study in that it increases the ecological validity of the study. Likewise, the individual interviews also contributes to the ecological validity of the study in that they provide additional details and insights behind the specific factors the teachers perceive as demanding.

In any event, and despite the longitudinal design, an important limitation of the study is that we only had two assessment rounds with a quite long time separation (i.e. on average 30 months). Observably, the study design is insensitive to finding, or tracking, potential changes and fluctuations that may occur between the two measurements. Further, the different scales and directions of the variables may to some extent make the interpretation of the explanatory variables (risk factors and protective factors) more difficult. Other limitations were that all data were self-reported, and that there was some statistical uncertainty with wide confidence intervals in the outcome measures.

There was only a minor difference in the frequencies of high burnout at baseline between the participants at follow-up and the total group of drop-outs. Thus, we do not believe that this overall selection of participation at follow-up have influenced the results to any major extent. However, a careful analysis in relation the various causes for non-participation suggest that a higher frequency of both the most affected and the healthiest teachers left the study, whereas the teachers with medium burnout levels at baseline remained at follow-up. For example, high burnout at baseline was more common among the group of drop-outs (*n* = 24) who were off duty/changed work, compared to those who participated at follow-up (37% vs. 14%). In contrast, among the teachers that were retired at follow-up (*n* = 43) only 4% reported high burnout at baseline. This selection may have influenced the results, but most likely towards less obvious patterns.

As shown when baseline data was stratified with respect to self-efficacy and job demands, which were the factors most strongly associated with burnout in the previously reported cross-sectional analysis [24], both low self-efficacy and high job demands co-occurred with a number of other factors. Thus, there is a risk of conceptual overlap between certain variables. Most pronounced, the three demand indicators (i.e. job demands, emotional demands and demands on hiding emotions) were correlated to an extent that only job demands were entered in the multivariate statistical analyses. The risk of potential confounding suggest that appropriate caution is warranted when interpreting the results.

The aim of the interviews was to complement the quantitative analysis with additional information, from many individuals, about their perception of the work environment. However, the interviews lasted for about ten minutes and cannot be considered as any in-depth interviews. Further, we did not conduct any recordings, but the interviewer took field notes. Thus, there is a need of a cautious interpretation of the results. Still, we had a large number of responders and the results gave a picture of underlying factors associated with the high job demands that many teachers experience. Large fractions of the teachers gave similar responses which made it possible to distinguish patterns and receive details of the exposure that was not captured by the questionnaire. Such information is valuable for guidance in preventive actions.

### Analytical considerations

In analyses of longitudinal studies, aiming to identify causal relationships, the most common method is to select only the participants who were healthy at baseline and study the exposures in baseline in relation to the onset of disease at follow up. However, such selection not only decreases statistical power but is also inappropriate if the aim is to study fluctuating health conditions where the investigated factors may not only influence the onset but also recovery from the conditions.

In contrast to traditional analyses of to which extent the exposure at baseline is of importance for the health status at follow-up, the conclusions regarding causal relationships from analyses of changes in exposure versus changes in outcome are weaker (the changes are measured simultaneously and by the study participants themselves). Still, in the light of the fluctuation among the teachers between the levels of burnout, the analyses give some interesting information of the factors associated with a changing work situation (in this case job demands and decision latitude) and changes in the teachers’ wellbeing.

### The results in relation to previous studies

The personal resources in terms of general self-efficacy turned out to be the strongest explanatory factor for burnout at follow-up, also after adjustment of the levels of burnout at baseline. Similar results have been reported earlier: Shoji et al. [33] found associations between job burnout and self-efficacy in a meta-analysis of studies in different occupations, Dicke et al. [15] detected direct effects of self-efficacy on emotional exhaustion in a longitudinal study among teachers, and Lauermann and König [34] found negative correlations between high self-efficacy and burnout (all three underlying dimensions in our burnout measure). Also, in the latter study [34] a specific teacher self-efficacy was identified, which was strongly associated with burnout. According to Schwarzer and Hallum [35] teacher self-efficacy is a personal resource that may protect from the experience of job strain and thus make an escalation of burnout less likely.

Although not a static concept, general self-efficacy is sometimes regarded as an inherent, or long lasting, quality that may differ among individuals. On the group level there were only minor differences in self efficacy between baseline and follow-up. However, on the individual level there was a substantial variation – in both directions - between baseline and follow-up. This probably reflects, as originally theorized by Bandura [36], that self-efficacy is dependent on the interplay between external and internal factors. Still, the perceived changes in general self-efficacy scores seem to be less important for determining changes in levels of burnout, compared to variations in job demands and decision latitude scores. To what extent this reflects that people in general are more likely to attribute changes as due to external conditions as opposed to attributing changes to alterations in one’s own personality or self-image is not known. One may suspect that individuals with low self-efficacy perceive higher job demands compared to those with a high self-efficacy. However, the correlation between the continuous variables job demand and self-efficacy was rather weak.

Perceived high job demands at baseline was of importance for burnout at follow up. However, in the last step when adjusting the multi-exposure model for the level of burnout at baseline, job demands was no longer a significant explanatory factor. This may be explained by the fact that there was a strong association between job demands and burnout already at baseline [24], and that there was no further increase of the association at follow up.

Our finding that high job demands was of importance for burnout is in line with several other studies (e.g. Aronsson et al. [8]). Further, increased job demands scores between baseline and follow-up was associated with an increased level of burnout. However, it was somewhat unexpected that we neither found an association between a low decision latitude and burnout in the cross-sectional study at baseline [24], and nor as a explanatory factor in the present follow-up study. Compared to other occupational groups such as nurses and sonographers [25], most of the teachers generally perceived rather high job control, and thus job control may not be the most crucial risk factor for burnout among them. However, in the analysis of *changes* in decision latitude versus burnout at follow-up, a decrease of decision latitude was associated with an increased level of burnout. More extensive explanations to these observations may be found in the interview-responses: many teachers perceive a continuous increase of new demands and work tasks, which may result in increased time pressure, reduced influence and less freedom to determine how the work is to be performed. Further, the teachers’ perception of not being able to do a good job and achieve their own pedagogical goals may contribute to increased burnout.

Fortunately, a major fraction of those with high burnout at baseline reported a better health at follow-up. The fluctuation between the levels of burnout indicate that for most of the teachers the level of burnout is not a static condition. Only one third of the teachers were without any burnout signs (level 0) at both baseline and follow-up and only 5% reported high burnout at both occasions. The remaining part of the study sample reported either a better or a worse level of burnout at follow up. However, in spite of the fluctuation in burnout on the individual level, at group level the burnout-status at baseline was of importance for the level of burnout at follow-up.

Beyond the observed associations with changes in job demands and decision latitude there may be other possible explanations to the changes of the teachers’ burnout, at both work and in private life, which were not captured in our study. For example, a previous study showed that imbalance between work and private life, i.e. too much work and too little free time for recovery and pleasure, predicted stress-related disorders [37].

### Possible implications

Our finding of a low self-efficacy as an explanatory factor for burnout indicate that actions that strengthen both individuals and the team/collective (collective efficacy [38, 39]), may have beneficial effects for the teacher’s well-being. However, to influence an inherent quality such as self-efficacy by organisational changes or political decisions is difficult. Further, the perceived changes in general self-efficacy scores seem to be less important for determining changes in levels of burnout, compared to variations in job demands and decision latitude scores.

There should be greater opportunities of preventing actions aiming to reduce the job demands. A contributing factor to the high job demands that teachers experience may be uncertainties in responsibilities and capacity/power. Thus, there is a need of clearer goals, both at national and local level, and a distribution of responsibilities that are in line with the goals. Support from school leaders in prioritizing between tasks and in assessing when a job is done well enough, may be other measures that reduce the workload. Further, the amount of different work tasks should be reduced, e g by increased, or better use of resources together with support from administrative staff.

A decrease in decision latitude may be a consequence of the high demands: failure to handle all work tasks due to high pressure may lead to a reduced opportunity to influence how work should be done, which in turn might lead to loss of control. Thus, measures to reduce the job demands may also have an impact on the perception of control.

## Conclusions

Many occupational, sociodemographic and life-styles factors and self-efficacy, as well as and the level of burnout at baseline, were of statistically significant importance for subsequent burnout two to three years later. Among the occupational and personal factors, job demands and self-efficacy were the strongest explanatory variables when all factors were analysed simultaneously. Noticeably, these two main explanatory variables were only weakly correlated with each other. That many teachers shifted level of burnout during the observation period underlines to some extent that burnout may have a cyclic pattern. Yet, the shifts in burnout were associated with changes in demands (increased) and control (decreased), but was not as much associated with changes in self-efficacy or with any other of the studied factors. Results from the qualitative analysis suggest that the teachers face a complex configuration of demands. Taken together, our findings suggest that a substantial proportion of the teachers have a problematic symptomatology that needs to be dealt with, via actions on individual, organizational and a societal levels.

## Additional files

Additional file 1: Interview guide, including the four open questions asked in the interview of the teachers. (DOC 26 kb)

Additional file 2: **Table S1.** With description of all occupational, sociodemographic and life-style factors and self-efficacy at baseline, stratified by the four levels of burnout at follow-up. (DOCX 17 kb)

## Abbreviations

MBI-GS: Maslach Burnout Inventory – General Survey

## References

[1] Sterling P, Allostasis EJ (1988). A new paradigm to explain arousal pathology. Handbook of life stress, cognition and health.

[2] Sterling P (2012). Allostasis: a model of predictive regulation. Physiol Behav.

[3] Ursin H, Eriksen HR (2004). The cognitive activation theory of stress. Psychoneuroendocrinology.

[4] Chrousos GP (2009). Stress and disorders of the stress system. Nat Rev Endocrinol.

[5] Johnson S, Cooper C, Cartwright S, Donald I, Taylor P, Millet C (2005). The experience of work-related stress across occupations. J Manag Psychol.

[6] AFA försäkring (AFA Insurance). Allvarliga arbetsskador och långvarig sjukfrånvaro. Afa försäkring, 2016 (in Swedish).

[7] Maslach C, Leiter MP (1997). The truth about burnout: how organizations cause personal stress and what to do about it.

[8] Aronsson G, Theorell T, Grape T, Hammarström A, Hogstedt C, Marteinsdottir I, Skoog I, Träskman-Bendz L, Hall C. A systematic review including meta-analysis of work environment and burnout symptoms. BMC Public Health. 2017;264. 10.1186/s12889-017-4153-7 PMC5356239 28302088

[9] Scott C, Stone B, Dinham S (2001). I Love Teaching but…. International Patterns of Teacher Discontent. Edu-cation Policy Analysis Archives..

[10] Brown M, Ralph S, Brember I (2005). Change-linked work related stress in British teachers. Res Educ.

[11] Travers CJ, Cooper CL (1996). Teachers under pressure: stress in the teaching profession.

[12] Naring G, Vlerick P, Van de Ven B (2011). Emotion work ¨ and emotional exhaustion in teachers: the job and individual perspective. Educ Stud.

[13] Bandura A (1977). Self-efficacy. Toward a unifying theory of behavioural change. Psychol Rev.

[14] Taris TW, Schaufeli WB, van Veldhaven M, Peccei R (2015). Individual well-being and performance at work: a conceptual and theoretical overview.

[15] Dicke T, Stebner F, Linninger C, Kunter M, Leutner D. A longitudinal study of teachers’ occupational well-being: applying the job demands-resources model. J Occup Health Psychol. 2017. 10.1037/ocp0000070 28150993

[16] Evers WJ, Brouwers A, Tomic W (2002). Burnout and self-efficacy: a study on teachers’ beliefs when implementing an innovative educational system in the Netherlands. Br J Educ Psychol.

[17] Duquett A, Kerouac S, Sandhu BK, Beaudet L (1994). Factors related to nursing burnout: a review of empirical knowledge. Issues Ment Health Nursb.

[18] Thompson CA, Prottas DJ (2006). Relationships among organizational family support, job autonomy, perceived control, and employee well-being. J Occup Health Psychol.

[19] Littrell PC, Billingsley BS, Cross LH (1994). The effects of principal support on special and general educators’ stress, job satisfaction, school commitment, health, and intent to stay in teaching. Remedial Spec Educ.

[20] Arvidsson I, Arvidsson M, Axmon A, Hansson G-Å, Johansson CR, Skerfving S (2006). Musculoskeletal disorders among female and male air traffic controllers performing identical and demanding computer work. Ergonomics.

[21] Gluschkoff K, Elovainio M, Kinnunen U, Mullola S, Hintsanen M, Keltikangas-Järvinen L, Hintsa T (2016). Work stress, poor recovery and burnout in teachers. Occup Med (London).

[22] Håkansson Carita, Ahlborg Gunnar (2017). Occupational imbalance and the role of perceived stress in predicting stress-related disorders. Scandinavian Journal of Occupational Therapy.

[23] Lindegård A, Jonsdottir IH, Börjesson M, Lindwall M, Gerber M (2015). Changes in mental health in compliers and noncompliers with physical activity recommendations in patients with stress-related exhaustion. BMC Psychiatry.

[24] Arvidsson I, Håkansson C, Karlson B, Björk J, Persson R (2016). Burnout among Swedish school teachers – a cross-sectional analysis. BMC Public Health.

[25] Arvidsson I, Gremark Simonsen J, Dahlqvist C, Axmon A, Karlson B, Björk J, Nordander C (2016). Cross-sectional associations between occupational factors and musculoskeletal pain in women teachers, nurses and sonographers. BMC Musculoskelet Disord.

[26] Maslach C, Jackson SE, Leiter MP. Maslach burnout inventory (3rd ed.). Palo Alto, CA: Consulting Psychologists Press; 1996.

[27] Maslach C, Jackson SMBI. Maslach burnout inventory ("human services survey"). Research edition. Manual. Palo Alto CA: Consulting Psychologists Press; 1981.

[28] Karasek R, Theorell T (1990). Healthy work stress, productivity and the reconstruction of working life.

[29] Karasek R, Brisson C, Kawakami N, Houtman I, Bongers P, Amick B (1998). The job content questionnaire (JCQ): an instrument for internationally comparative assessments of psychosocial job characteristics. J Occup Health Psychol.

[30] Kristensen TS, Hannerz H, Hogh A, Borg V (2005). The Copenhagen psychosocial questionnaire - a tool for the assessment and improvement of the psychosocial work environment. Scand J Work Environ Health.

[31] Persson R, Cleal B, Øllgaard Jacobsen M, Villadsen E, Andersen L (2014). The relationship between self-efficacy and help evasion. Health Educ Behav.

[32] Graneheim UH, Kundman B (2004). Qualitative content analysis in nursing research: concepts, procedures and measures to achieve trustworthiness. Nurse Educ Today.

[33] Shoji K, Cieslak R, Smuktunowicz E, Rogala A, Benight C, Luszczynska A (2016). Associations between job burnout and self-efficacy: a meta-analysis. Anxiety, stress & coping.

[34] Lauermann F, König J (2016). Teachers professional competence and wellbeing: understanding the links between general pedagogical knowledge, self-efficacy and burnout. Learn Instr.

[35] Schwarzer R, Hallum S (2008). Perceived teacher self-efficacy as a predictor of job stress and burnout: mediation analysis. Appl Psychol.

[36] Bandura Albert (1978). The self system in reciprocal determinism. American Psychologist.

[37] Håkansson C, Ahlborg G (2018). Occupational imbalance and the role of perceived stress in predicting stress-related disorders. Scand J Occup Ther.

[38] Bandura A (1997). Self-efficacy: the exercise of control.

[39] Goddard RD (2001). Collective efficacy: a neglected construct in the study of schools and student achievement. J Educ Psychol.

