# Supplementary material for: Burnout among school teachers: quantitative and qualitative results from a follow-up study in southern Sweden
Source: BMC Public Health. 2019 May 29;19:655. doi: 10.1186/s12889-019-6972-1 (PMC6542045; doi:10.1186/s12889-019-6972-1)
Supplement: Supplementary file 1 — Interview guide, including the four open questions asked in the interview of the teachers. (DOC 26 kb) [file 12889_2019_6972_MOESM1_ESM.doc]

INTERVIEW GUIDE

1. Which favourable work conditions do you perceive?
2. Which unfavourable work conditions do you perceive?
3. Which work tasks do you perceive as ergonomically stressful?
4. Do you have any suggestions of improvements of the work environment at your workplace?
