# Supplementary material for: Burnout among school teachers: quantitative and qualitative results from a follow-up study in southern Sweden
Source: BMC Public Health. 2019 May 29;19:655. doi: 10.1186/s12889-019-6972-1 (PMC6542045; doi:10.1186/s12889-019-6972-1)
Supplement: Supplementary file 2 — Table S1. With description of all occupational, sociodemographic and life-style factors and self-efficacy at baseline, stratified by the four levels of burnout at follow-up. (DOCX 17 kb) [file 12889_2019_6972_MOESM2_ESM.docx]

**Supplementary table S1.** Occupational, sociodemographic- and life-style factors and self-efficacy *at baseline*, among 310 teachers (230 females and 80 males), stratified into four levels of burnout at *follow-up;* level 0 (no burnout in any dimension), level 1 (burnout in one dimension), level 2 (burnout in two dimensions) and level 3 (burnout in all three dimension).

|  |  |  |  | **Burnout at follow-up** | | | |  |
| --- | --- | --- | --- | --- | --- | --- | --- | --- |
|  | *Scale* | N | All | Level 0 | Level 1 | Level 2 | Level 3 | Test for trend |
| **Characteristics at baseline** |  |  |  | N=143 | N=119 | N=38 | N=10) | p |
|  |  |  |  |  |  |  |  |  |
| *Year of compulsory school* |  | 310 |  |  |  |  |  | 0.08 |
| year 4-6 ; n (%) |  |  | 100 (32) | 53 (37) | 35 (29) | 10 (26) | 2 (20) |  |
| year 7-9 ; n (%) |  |  | 210 (68) | 90 (63) | 84 (71) | 28 (74) | 8 (80) |  |
|  |  |  |  |  |  |  |  |  |
| Seniority; mean (SD) | *years* | 310 | 17 (11) | 18 (12) | 17 (11) | 15 (11) | 13 (8.3) | 0.11 |
|  |  |  |  |  |  |  |  |  |
| Complaints on computer |  |  |  |  |  |  |  |  |
| workstation arrangements; mean (SD) | *1-5^a^* | 306 | 3.1 (1.1) | 2.9 (1.1) | 3.1 (1.1) | 3.4 (1.0) | 3.2 (1.3) | 0.02 |
| Job demands; mean (SD) | *1-4^a^* | 308 | 2.9 (0.4) | 2.8 (0.4) | 3.0 (0.4) | 3.1 (0.4) | 3.1 (0.3) | <0.001 |
| Job control – decision latitude; mean (SD) | *1-4^b^* | 310 | 3.1 (0.4) | 3.1 (0.4) | 3.0 (0.5) | 3.1 (0.4) | 3.1 (0.6) | 0.64 |
| Job control - skill discretion; mean (SD) | *1-4^b^* | 310 | 3.4 (0.3) | 3.4 (0.3) | 3.4 (0.3) | 3.3 (0.3) | 3.3 (0.5) | 0.18 |
| Job support from manager; mean (SD) | *0-4^b^* | 310 | 2.6 (0.5) | 2.8 (0.5) | 2.6 (0.5) | 2.4 (0.5) | 2.4 (0.7) | <0.001 |
| Job support from collegues; mean (SD) | *0-4^b^* | 308 | 3.1 (0.4) | 3.1 (0.4) | 3.1 (0.4) | 2.9 (0.4) | 3.0 (0.5) | 0.10 |
| Emotional demands; mean (SD) | *0-4^a^* | 310 | 2.8 (0.7) | 2.6 (0.7) | 2.9 (0.7) | 3.0 (0.7) | 2.9 (0.6) | <0.001 |
| Demands of hiding emotions; mean (SD) | *0-4^a^* | 310 | 1.7 (0.8) | 1.6 (0.7) | 1.7 (0.8) | 2.0 (0.8) | 1.8 (0.8) | 0.01 |
| Leadership; mean (SD) | *0-4^b^* | 310 | 2.1 (0.8) | 2.2 (0.8) | 2.0 (0.8) | 1.7 (0.8) | 1.7 (0.9) | <0.001 |
| Self- efficacy; mean (SD) | *1-5^b^* | 309 | 4.1 (0.5) | 4.2 (0.5) | 4.0 (0.5) | 3.9 (0.6) | 4.0 (0.3) | <0.001 |
|  |  |  |  |  |  |  |  |  |
| Gender |  | 310 |  |  |  |  |  | 0.69 |
| Men; n (%) |  |  | 80 (26) | 35 (24) | 33 (28) | 8 (21) | 4 (40) |  |
| Women; n (%) |  |  | 230 (74) | 108 (76) | 86 (72) | 30 (79) | 6 (60) |  |
|  |  |  |  |  |  |  |  |  |
| *Marital status* |  | 306 |  |  |  |  |  | 0.87 |
| Married/cohabitant |  |  | 264 (87) | 118 (86) | 107 (90) | 31 (84) | 8 (80) |  |
| Single |  |  | 40 (13) | 20 (14) | 12 (10) | 6 (16) | 2 (20) |  |
|  |  |  |  |  |  |  |  |  |
| Age, years; mean (SD) | *years* | 310 | 47 (10) | 47 (11) | 47 (11) | 47 (10) | 44 (8.4) | 0.33 |
| Personal relaxation time; mean (SD) | *1-6^b^* | 304 | 3.4 (1.3) | 3.7 (1.3) | 3.3 (1.2) | 3.1 (1.4) | 3.0 (1.1) | 0.002 |
| Household work; mean (SD) | *1-5^a^* | 306 | 2.9 (0.8) | 2.8 (0.8) | 3.0 (0.9) | 2.7 (0.8) | 3.2 (0.9) | 0.001 |
| Physical exercise; mean (SD) | *0-4^b^* | 307 | 2.6 (1.2) | 2.8 (1.0) | 2.5 (1.2) | 2.3 (1.3) | 2.0 (1.5) | 0.47 |
|  |  |  |  |  |  |  |  |  |
| ***Original MBI-GS dimensions, at follow up*** |  |  |  |  |  |  |  |  |
| *Exhaustion* | 0-6 | 310 | 3.0 (1.5) | 1.9 (0.9) | 3.7 (1.2) | 4.5 (0.8) | 5.0 (0.5) |  |
| *Cynicism* | 0-6 | 310 | 1.7 (1.3) | 1.1 (0.9) | 1.7 (0.9) | 3.5 (1.3) | 4.5 (0.7) |  |
| *Professional efficacy* | 0-6 | 310 | 5.1 (0.7) | 5.4 (0.5) | 4.9 (0.7) | 4.6 (0.8) | 3.8 (0.5) |  |
|  |  |  |  |  |  |  |  |  |
|  |  |  |  |  |  |  |  |  |

^a^Higher scores indicate a more unfavourable situation.

^b^ Higher scores indicate a more favourable situation.
